# Effect of cost-reduction interventions on facility-based deliveries in Burkina Faso: a controlled interrupted time-series study with multiple non-equivalent dependent variables

**DOI:** 10.1136/jech-2022-218794

**Published:** 2022-12-20

**Authors:** Ivlabèhiré Bertrand Meda, Seni Kouanda, Valéry Ridde

**Affiliations:** 1 Département Biomédical, Institut de Recherche en Sciences de la Santé, Ouagadougou, Burkina Faso; 2 Institut Africain de Santé Publique (IASP), Ouagadougou, Burkina Faso; 3 Centre Population et Développement (CEPED), Inserm, IRD, Université Paris Cité, F-75006 Paris, France

**Keywords:** EPIDEMIOLOGY, HEALTH POLICY, PUBLIC HEALTH, STUDY DESIGN

## Abstract

**Background:**

Evaluating health intervention effectiveness in low-income countries involves many methodological challenges to be addressed. The objective of this study was to estimate the sustained effects of two interventions to improve financial access to facility-based deliveries.

**Methods:**

In an innovative controlled interrupted time-series study with primary data, we used four non-equivalent dependent variables (antenatal care) as control outcomes to estimate the effects of a national subsidy for deliveries (January 2007–December 2013) and a local ‘free delivery’ intervention (June 2007–December 2010) on facility-based deliveries. The statistical analysis used spline linear regressions with random intercepts and slopes.

**Results:**

The analysis involved 20 877 observations for the national subsidy and 8842 for the ‘free delivery’ intervention. The two interventions did not have immediate effects. However, both were associated with positive trend changes varying from 0.21 to 0.52 deliveries per month during the first 12 months and from 0.78 to 2.39 deliveries per month during the first 6 months. The absolute effects, evaluated 84 and 42 months after introduction, ranged from 2.64 (95% CI 0.51 to 4.77) to 10.78 (95% CI 8.52 to 13.03) and from 9.57 (95% CI 5.97 to 13.18) to 14.47 (95% CI 10.47 to 18.47) deliveries per month for the national subsidy and the ‘free delivery’ intervention, respectively, depending on the type of antenatal care used as a control outcome.

**Conclusion:**

The results suggest that both interventions were associated with sustained non-linear increases in facility-based deliveries. The use of multiple control groups strengthens the credibility of the results, making them useful for policy makers seeking solutions for universal health coverage.

**WHAT IS ALREADY KNOWN ON THIS TOPIC**

- Previous studies that have evaluated the sustained effects of interventions to reduce or eliminate fees for access to delivery care are rare, and the results are inconsistent.
- Most of this research has assumed that facility-based deliveries follow a linear trend and that interventions have linear effects.

**WHAT THIS STUDY ADDS**

- This study shows that a local intervention without any external support can have greater sustained effects on facility-based deliveries than a national subsidy policy.
- The study shows that the trends in facility-based deliveries and the effects of interventions are not always linear.

**HOW THIS STUDY MIGHT AFFECT RESEARCH, PRACTICE OR POLICY**

- In the absence of intervention-free groups to serve as controls, alternatives such as non-equivalent dependent variables may be availed to increase the robustness of the results.

## Introduction

As part of a global commitment to improve maternal health and reduce maternal and neonatal mortality, sub-Saharan African countries have introduced policies to increase financial access to delivery care.^1^ Several studies have supported that these interventions are associated with an increase in institutional deliveries,^2–15^ although systematic reviews have highlighted the methodological weaknesses of many of these studies.^16 17^

Nonetheless, studies that have estimated the effects of these interventions beyond 3 years remain limited, and the results are inconsistent.^3–5 7 11 12 14^ The most reliable evidence has produced one difference-in-differences design^7^ and four interrupted time-series studies,^4 5 11 12^ only one of which used a control group.^12^ The fact that such interventions are often introduced at the national level from the outset makes it impossible to establish control groups.

Additionally, almost all previous time-series studies^4 5 11^ have assumed that facility-based deliveries follow a linear progression and that the effects of interventions are also linear. However, institutional deliveries are influenced by factors that can vary rapidly, sometimes within the same year. This is the case for several factors that influence the perceived quality of care, including the availability of equipment, supplies and drugs; the availability and friendliness of staff; and health workers’ leadership.^18 19^ Additionally, several studies have shown that the implementation of these interventions has often been affected by reimbursement delays, insufficient funding, unavailability of drugs, etc^20–22^; therefore, their implementation is rarely linear. Some authors believe that linear trends are relatively rare among the outcomes targeted by health interventions^23^; thus, a linear approach can lead to biased estimates of the intervention effects.

The possibility of rapid change in the trend of facility-based deliveries calls into question the use of the preintervention trend as a counterfactual in uncontrolled interrupted time-series studies. Moreover, this approach does not ensure that untreated controls are always similar to the intervention groups in terms of characteristics correlated with outcome throughout the entire observation period.

All these methodological difficulties have led some authors to advocate the use of non-equivalent dependent variables rather than untreated comparison groups.^23–25^ A non-equivalent dependent variable (control outcome) is an outcome that is not expected to respond to the intervention but that would react to potential confounding factors that would also affect the outcome of interest. In addition to offering the same advantages as an untreated control group, this approach allows us to control for local history bias and to reduce the risk of selection bias, as the intervention and control groups share the same contextual factors and important determinants of health outcomes.^23–25^ Unfortunately, this methodological approach has not yet been widely used in the evaluation of health interventions. We did not find any studies using this approach to evaluate interventions intended to improve financial access to health services in sub-Saharan Africa.

The objective of this study is to estimate the sustained effects of two interventions to improve financial access to facility-based deliveries in Burkina Faso. Its originality is threefold: (1) the use of multiple (four) non-equivalent dependent variables rather than untreated control groups, (2) the use of primary data versus the secondary data used in time-series analyses and (3) a non-linear approach to natural trends and intervention effects.

## Methods

### Context and interventions

This study evaluates two interventions that differed in the extent to which they reduced user fees and were implemented in two neighbouring districts. First, a national subsidy for deliveries and emergency obstetric care (EmONCs) was introduced between January and June 2007 depending on the health district. It has been widely described elsewhere.^14 18^ Briefly, it was a cost-sharing system in which the state paid 80% of the direct medical expenses for deliveries and EmONCs and households paid the remaining 20%. In June 2016, this policy was replaced by a policy of free care for women and children under 5. This intervention has been evaluated for the Zorgho district. At the end of 2013, this district had 50 primary health centres, called ‘Centres de Santé et de Promotion Sociale’ (CSPSs), and one district hospital.

In conjunction with this national policy introduced in June 2007, the Kaya district paid, from its decentralised annual budget, the 20% of direct medical expenses that were not covered by the national subsidy, making deliveries free of charge. This ‘free delivery’ intervention has also been described elsewhere.^26^ The particularity of this intervention is that it had no external support or funding, unlike similar interventions in other districts.^12 27^ In early 2011, the district discontinued this ‘free delivery’ intervention and reverted to the national subsidy because the financial resources had become insufficient given the increase in deliveries in CSPSs. This funding shortage was exacerbated by delays in reimbursement of the national subsidy by the Ministry of Health.^26^ No research on the effects of this intervention on service utilisation have yet been published. The Kaya district had 48 CSPSs at the end of 2010.

### Study design

The study is a controlled interrupted time-series design in which the controls were non-equivalent dependent variables. The observation periods are from January 2005 to December 2013 for Zorgho and from January 2006 to December 2010 for Kaya. The time-series data are monthly.

The non-equivalent dependent variables consist of antenatal care (ANC) visits observed in the same facilities during the same periods. Facility-based deliveries and ANC share many common determinants, including sociocultural factors, economic and physical accessibility, and perceived benefit/need.^19 28 29^ Additionally, the use of ANC can increase delivery service use because it familiarises women with services and provides staff with an opportunity to promote assisted deliveries.^19 30^ However, we are not aware of any literature arguing that institutional deliveries influence the use of ANC. ANC has been free since 2002 at the national level,^9^ and it is therefore not expected to react to these two interventions. However, it is expected to respond to other changes (eg, population growth, type and number of providers, quality of care) that could also affect the trend in deliveries.^23^ Finally, Wagenaar *et al* 23 suggested that ANC could be used as a non-equivalent dependent variable to evaluate interventions that increase financial access to deliveries.

Four different types of ANC (ANC1, ANC2, ANC3 and ANC4) representing the monthly number of women who attended for their first, second, third and fourth or more antenatal visits, respectively, were included as control outcomes. It is likely that the different ANCs are highly correlated. However, in several sub-Saharan countries, pregnant women are reported to attend ANC to avoid being reprimanded by staff during delivery.^28^ Therefore, we can hypothesise that women who would like to use free or subsidised deliveries may use ANC to obtain the card and avoid reprimands at delivery. In this case, it is possible for both interventions to induce an increase in ANC use, but it is unlikely to affect all ANCs, including frequent visits. We included all four ANCs to ensure that the choice of type of ANC did not affect the conclusions and to increase the robustness of the results. This approach also increases the statistical power by expanding the number of observations.^25^

The observation period starts in January 2006 in Kaya because ANC data are available beginning on that date. The district hospital and the urban CSPS in Zorgho were excluded because they were not distinct institutions until 2009, and the inclusion of the hospital could lead to a double counting of the women served by the peripheral CSPSs.

### Data sources

The delivery data are primary data that were extracted from the facility’s daily delivery register between October 2014 and March 2015. All registers of deliveries and ANC visits for the study period from the 98 CSPSs in the two districts were collected at the district office by one of the authors. The opening date of each health facility was collected to ensure the exhaustiveness of the registers. When we visited each CSPS, we confirmed the start date of the intervention and the non-existence of other similar interventions. Each woman recorded in the register was then entered (using double entry) into an EPIDATA mask by medical students who had been trained for 2 days and were familiar with the registers. The input mask included the district, the CSPS, the woman’s age, the date of delivery and the weight and vital status of the newborn. We then cross-checked the data from the double entry to ascertain any inconsistencies. Finally, the data were aggregated by health facility and month.

The ANC data are secondary data obtained from the health information system (HIS) for each district. Several studies have already attested to the validity and reliability of the HIS data in Burkina Faso.^6 27 31 32^ However, to confirm the quality of these secondary data, we randomly selected 1 year and one type of ANC per facility and collected primary data from the registers for comparison with the secondary data. This data collection was carried out in the same way as for the delivery data.

### Study variables

#### Outcome variables

Our dependent variables were the monthly number of deliveries and ANC visits. For deliveries, the annual average in the primary data from each health facility was compared with that obtained from the HIS with Student’ s t-test, and any statistically significant differences (p<0.05) led to a check and a correction, if necessary, based on the register data. For ANC visits, the same tests were performed for the samples drawn. The statistical tests did not show any significant differences between the primary and secondary data.

#### Independent variables

We included the usual variables that are essential for a time trend analysis.^33 34^ In each district, we defined a group variable with five categories to identify deliveries and each category of ANC. A policy variable was created to represent the national subsidy in Zorgho (coded 1 for January 2007 onwards and 0 before) and “free deliveries” in Kaya (1 for June 2007 onwards and 0 before). The time variable was sequentially coded to indicate the months since the start of the observation periods in each district. The use of non-equivalent dependent variables as control outcomes renders the use of confounding variables unnecessary because any change over time in these variables (population growth, number of health workers, opening of new CSPSs, leadership of health workers, gender of the professional, etc) are expected to affect both ANC visits and deliveries.

### Statistical analysis

We first performed a descriptive analysis and searched for extreme values by health facility through the approach suggested by Shapira *et al*.^35^ We then performed spline linear regressions with mixed effects to estimate the effects of each intervention on facility-based deliveries. As the health facilities are predominantly rural and the geographical areas are relatively small, we assumed that the introduction of each intervention would result in an intercept jump and a slope change. We also assumed that the preintervention and postintervention trends were non-linear and chose to allow each calendar year to have its own slope. Intercepts and slopes were assumed to vary across health facilities. These assumptions led to the equation below:

$$Y_{it}=\begin{array}{l} \beta_{0i}+\beta_{1j}Group_{j}+\left(\sum_{y=1}^{k}\beta_{iy}Spline_{yt}\right)+\left(\sum_{m=2}^{12}\beta_{m}Month\right)+\\ \left(\sum_{y=1}^{k}\beta_{jy}Spline_{yt}*Group_{j}\right)+\beta_{Ei}Policy+\beta_{Ej}Policy*Group_{j}+e_{it} \end{array}$$

In this model, $Y_{it}$ represents the monthly number of users of health facility i at time t. $\beta_{0i}$ is the random intercept for delivery in each facility. $\beta_{1j}$ is the difference between the intercept for the delivery outcome and that for ANC outcome j. $\beta_{iy}$ is the random coefficient for delivery in facility i attached to segment yt. $\beta_{jy}$ is the difference in the slope between the delivery outcome and ANC outcome j. Month is a dummy variable representing the calendar month and is used to adjust for seasonal variation. $\beta_{Ei}$ represents the immediate effect of each intervention at facility i, while $\beta_{Ej}$ is the difference in the immediate effect between the delivery outcome and ANC outcome j.

Using the full model, we first compared the preintervention slopes for the different outcomes. In the Kaya district, ANC4 had a slope that differed significantly from the others and was therefore dropped from the analysis. We then compared the successive slopes for each outcome and removed knots separating those slopes that were not significantly different for any outcome. In addition, we estimated a single slope when that slope did not differ between outcomes within a segment. Rather than using time segments, time was introduced as a random effect to prevent model convergence problems.^36^ A first-order autoregressive (AR (1)) structure stratified by group was used to account for heteroskedastic level-1 residuals. Comparisons between models were made with the Akaike information criterion and the Bayesian information criterion.

From the parsimonious model, we took differences to estimate the effects of each intervention on the postintervention slope. The absolute effects of each intervention over time were estimated by assuming that deliveries would have progressed in a manner similar to that of ANC in the absence of the intervention.

All analyses were conducted with the mixed function in Stata V.15.2 (StataCorp), and the significance level of the statistical tests was set to 0.05.

## Results

Seven CSPSs, which opened in 2010, a few months before the abolition of the ‘free delivery’ intervention, were excluded from the analysis in the Kaya district because they did not apply this intervention. In the Zorgho district, one CSPS that opened in 2013 and had fewer than six observations was also excluded. The analysis included 20 877 and 8842 observations for 21 085 and 8948 expected in Zorgho and Kaya, respectively. The rates of missing data were 1% in Zorgho and 1.2% in Kaya (online supplemental table 1). The number of users increased postintervention for all types of outcomes in both districts (table 1).

10.1136/jech-2022-218794.supp1 Supplementary data

**Table 1:** Descriptive statistics for the outcome variable (deliveries) and the non-equivalent dependent variables (antenatal care (ANC)) in the two districts before and after introduction of the interventions

	Kaya health district				Zorgho health district				
	Deliveries	ANC1	ANC2	ANC3	Deliveries	ANC1	ANC2	ANC3	ANC4
No of facilities	41	41	41	41	48	48	48	48	48
No of observations in the preintervention period	563	557	556	559	766	763	761	762	762
Mean monthly users in the preintervention period (SD)	17.3 (15.1)	43.1 (24.5)	33.8 (18.5)	20.6 (13.5)	18.8 (11.5)	35.3 (18.8)	28.3 (14.9)	16.2 (10.1)	5.5 (5.2)
No of observations in the postintervention period	1667	1639	1650	1651	3448	3405	3418	3409	3383
Mean monthly users in the postintervention period (SD)	27.5 (17.0)	45.7 (29.2)	38.9 (22.7)	26.9 (15.8)	26.6 (15.8)	33.0 (19.8)	30.0 (17.7)	22.9 (14.0)	12.4 (9.8)

ANC1: first ANC visit, ANC2: second ANC visit, ANC3: third ANC visit, ANC4: fourth ANC visit.

SD, standard deviation.

At the onset of the study period, the numbers for each type of ANC were significantly different from those for deliveries except for that of ANC3 (p=0.724 in Kaya and p=0.338 in Zorgho). For example, the average number of ANC1 was 33.19 vs 9.66 deliveries in Kaya. In both districts, the preintervention trends were similar for deliveries and ANC. The number of deliveries and ANC visits increased monthly on average by 0.47 in Kaya and 0.16 in Zorgho (online supplemental table 2).

10.1136/jech-2022-218794.supp2 Supplementary data

The introduction of the intervention did not lead to an immediate increase for deliveries in either Kaya (coefficient=−0.32, p=0.736) or Zorgho (coefficient=−0.13, p=0.821). However, there was an immediate and significant increase of 10.3 ANC1 and 5.0 ANC2 following the introduction of the ‘free delivery’ intervention in Kaya (online supplemental table 2).

Table 2 presents the immediate effect and the change in facility-based deliveries monthly trend associated with the two interventions, depending on the type of ANC used as a control outcome.

**Table 2:** Immediate effect and change in trend on monthly facility-based deliveries associated with two cost-reduction interventions in Burkina Faso with different non-equivalent dependent variables as control outcomes

	Non-equivalent dependent variables as control outcomes			
	ANC1	ANC2	ANC3	ANC4
Kaya Health District				
Immediate intervention effect	−10.60 (−15.95 to −5.25)	−5.36 (−8.83 to −1.88)	−3.09 (−7.07 to 0.89)	
Change in monthly trend attributable to the intervention				
June –December 2007 trend	2.39 (1.71 to 3.08)	1.23 (0.72 to 1.75)	0.78 (0.22 to 1.35)	
January 2008 –December 2009 trend	0	0	0	
January –December 2010 trend	0.76 (0.44 to 1.07)	0.69 (0.40 to 0.98)	0.65 (0.41 to 0.89)	
Zorgho Health District				
Immediate intervention effect	0	0	0	0
Change in monthly trend attributable to the intervention				
January –December 2007 trend	0.52 (0.31 to 0.73)	0.31 (0.11 to 0.52)	0.21 (0.01 to 0.40)	0.46 (0.26 to 0.65)
January –December 2008 trend	0.005 (−0.20 to 0.21)	−0.03 (−0.23 to 0.17)	−0.18 (−0.37 to 0.005)	−0.33 (−0.53 to −0.14)
January–December 2009 trend	0.37 (0.20 to 0.55)	0.32 (0.15 to 0.49)	0.20 (0.04 to 0.36)	0.13 (−0.03 to 0.30)
January–December 2010 trend	0	0	0	0
January–December 2011 trend	0	0	0	0
January 2012–December 2013 trend	0	0	0	0

ANC1 (first ANC visit), ANC2 (second ANC visit), ANC3 (third ANC visit), ANC4 (fourth ANC visit). In each column, we have the effects of the intervention on facility-based deliveries when the type of ANC is used as a control. Data are shown as estimates (95% CI).

ANC, antenatal care.

During the first 7 months after the introduction of ‘free delivery’ in Kaya (June–December 2007), the changes in the facility-based deliveries trends associated with the ‘free delivery’ intervention were 0.78 (95% CI 0.22 to 1.35), 1.23 (95% CI 0.72 to 1.75) and 2.39 (95% CI 1.71 to 3.08) deliveries, depending on whether ANC3, ANC2 or ANC1 was used as the control outcome. Between eight and 32 months after intervention introduction (January 2008 to December 2009), there was no change on monthly trend of deliveries associated with the intervention. Finally, in 2010, ‘the free delivery’ intervention was also associated with increases in the trends of deliveries of 0.65 (95% CI 0.41 to 0.89), 0.69 (95% CI 0.40 to 0.98) or 0.76 (95% CI 0.44 to 1.07), depending on whether the control outcome was ANC3, ANC2 or ANC1.

**Figure 1:**
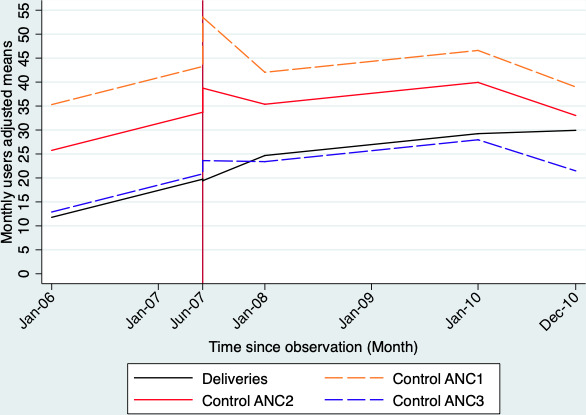
Plot of the estimated trends in deliveries and different types of antenatal care before and after the introduction of the “free delivery” intervention in the Kaya health district.

**Figure 2:**
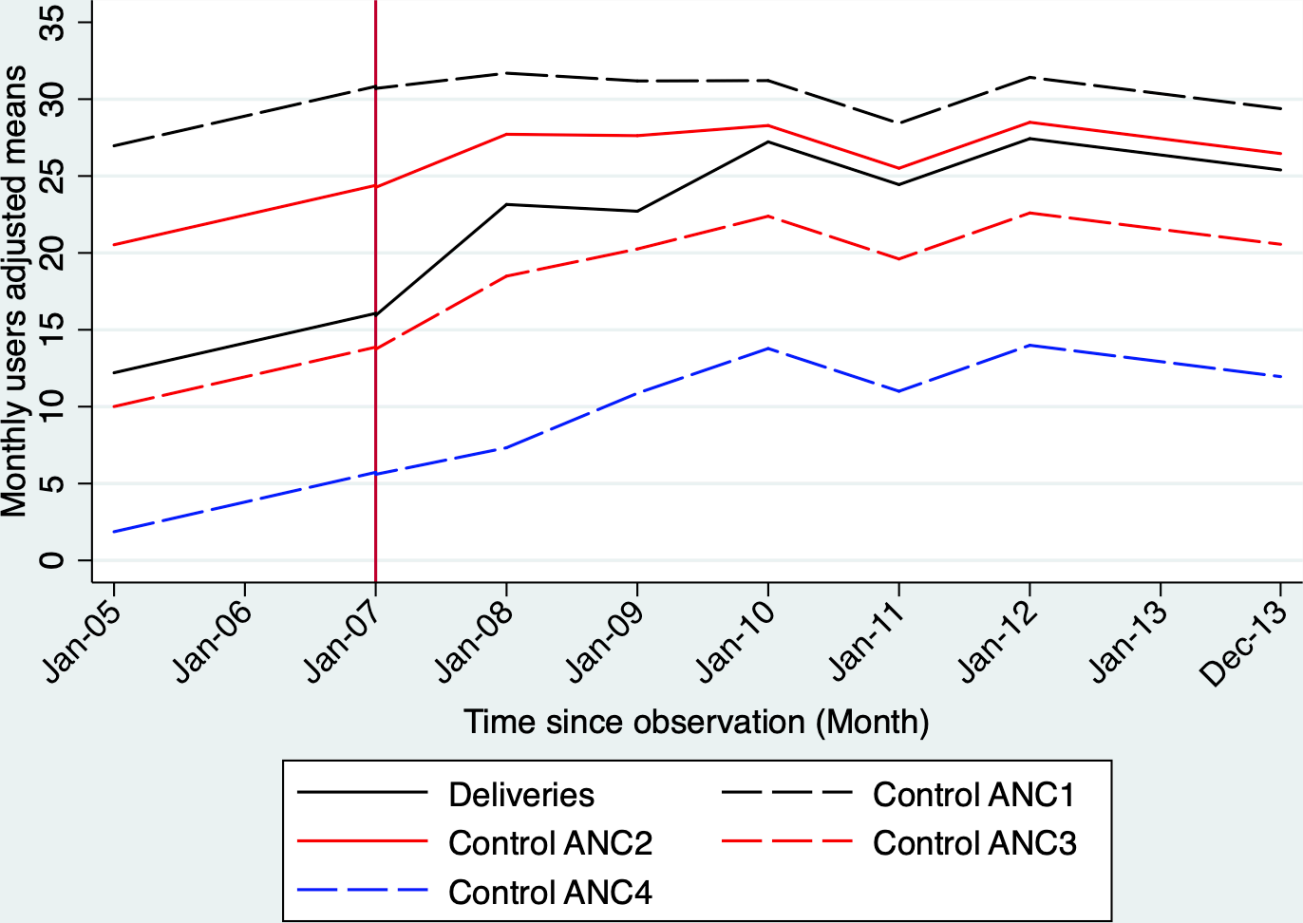
Plot of the estimated trends in deliveries and different types of antenatal care (ANC) before and after the introduction of the national subsidy policy for deliveries in the Zorgho health district.

In the Zorgho district, during the first 12 months following the introduction of the national subsidy (January to December 2007), the national subsidy was associated with significant increases in the trends of deliveries of 0.21 (95% CI 0.01 to 0.40), 0.31 (95% CI 0.11 to 0.52), 0.46 (95% CI 0.26 to 0.65) or 0.52 (95% CI 0.31 to 0.73) deliveries per month, depending on whether ANC3, ANC2, ANC4 or ANC1 was used as the control outcome. From the 13th to the 24th month after the introduction of the intervention (January to December 2008), the policy was associated with a decrease of 0.33 deliveries per month (95% CI −0.52 to −0.14) when ANC4 was used as a control, and the effects were null when other ANCs were considered as a control. From the 25th to the 36th month after the start of the national subsidy (January to December 2009), the intervention was associated with increases in the trends of deliveries of 0.20 (95% CI 0.04 to 0.36), 0.32 (95% CI 0.15 to 0.49) or 0.37 (95% CI 0.20 to 0.55), depending on whether ANC3, ANC2 or ANC1 was used as the control outcome. When ANC4 was used as the control outcome, the increase in the trend was not statistically significant (coefficient=0.13, 95% CI −0.03 to 0.30). Figure 1 (Kaya District) and figure 2 (Zorgho district) graphically present the trends for the deliveries and ANC.

Table 3 presents the absolute effects of the two interventions during different postintervention periods according to the type of ANC used as the control outcome. Regardless of the type of ANC used, the absolute effects of the “free delivery” intervention increased over time in Kaya. The effects of the national subsidy in Zorgho were less uniform across the different types of ANC considered but remained positive throughout the observation period.

**Table 3:** Absolute long-term effects with corresponding 95% CI: the effects of two cost-reduction interventions on facility-based deliveries in two districts in Burkina Faso with different non-equivalent dependent variables as control outcomes

Effects of the policy	ANC1	ANC2	ANC3	ANC4
Kaya Health District (Free Delivery Intervention)				
Immediate	−10.60 (−15.95 to −5.25)	−5.36 (−8.83 to −1.88)	−3.09 (−7.07 to 0.89)	
6 months	3.76 (0.33 to 7.19)	2.04 (−1.16 to 5.24)	1.60 (−1.27 to 4.48)	
12 months	6.16 (2.66 to 9.66)	3.28 (−0.15 to 6.71)	2.39 (−0.65 to 5.43)	
18–30 months	6.13 (2.64 to 9.61)	3.16 (− 0.20 to 6.53)	2.25 (−0.78 to 5.29)	
36 months	9.94 (6.63 to 13.24)	6.73 (3.33 to 10.13)	5.65 (2.61 to 8.69)	
42 months	14.47 (10.47 to 18.47)	10.88 (6.78 to 14.98)	9.57 (5.97 to 13.18)	
Zorgho Health District (National Subsidy)				
Immediate	0	0	0	0
6 months	3.11 (1.87 to 4.36)	1.88 (0.66 to 3.10)	1.23 (0.08 to 2.38)	2.74 (1.59 to 3.89)
12 months	6.22 (3.74 to 8.71)	3.76 (1.33 to 6.20)	2.46 (0.17 to 4.76)	5.48 (3.18 to 7.79)
18 months	6.26 (4.30 to 8.21)	3.59 (1.67 to 5.51)	1.36 (−0.46 to 3.18)	3.49 (1.66 to 5.32)
24 months	6.28 (4.14 to 8.44)	3.41 (1.31 to 5.52)	0.26 (−1.72 to 2.24)	1.50 (−0.52 to 3.52)
30 months	8.53 (6.59 to 10.48)	5.34 (3.42 to 7.25)	1.45 (−0.38 to 3.27)	2.30 (0.45 to 4.15)
36 to 84 months	10.78 (8.52 to 13.03)	7.26 (5.04 to 9.48)	2.64 (0.51 to 4.77)	3.10 (0.94 to 5.26)

ANC, antenatal care.

## Discussion

The results show that both interventions were associated with an increase in facility-based deliveries and that this increase persisted throughout the observation period, that is, 42 months for the ‘free delivery’ intervention in Kaya and 84 months for the national subsidy in Zorgho. However, this increase was not linear over time. Moreover, the effects of the ‘free delivery’ intervention appeared to be stronger than those of the national subsidy.

Previous studies have also reported an increase in facility-based deliveries associated with the national subsidy policy^5 8 11 12^ or district programmes for free delivery.^12^ Some of these studies^5 12^ have also shown that the effects were sustained over time, up to 72 months in the case of the national subsidy and up to 96 months for a removal of user fees in two districts of the Sahel region.^12^ However, the intervention in the Sahel region was led by a Non-Governmental Organization (NGO) and included other components (eg, staff training, equipment, supervision of activities) that could improve fidelity to the intervention, quality of care and provision.

The results also show that the effects of the ‘free delivery’ intervention were greater than those of the national subsidy. Earlier studies have also argued that the full removal of direct medical expenses was associated with a greater increase in facility-based deliveries than the partial subsidy.^12^ This finding suggests that the remaining 20% of direct medical expenses borne by patients remained a financial barrier for some households. In June 2016, Burkina Faso moved from the national subsidy to a policy of free maternal healthcare, although this new policy has yet to be evaluated.

We did not investigate the effects of these increases in facility-based deliveries on health outcomes (eg, maternal and neonatal mortality), which are the ultimate goals of these interventions. However, several studies^7 10 11^ have shown that these increases in delivery care use were not accompanied by improvements in health outcomes, suggesting that the financial barrier is not the only obstacle to improving maternal health in sub-Saharan Africa.

The innovations in this study are mainly methodological. To our knowledge, this is the first study to use non-equivalent dependent variables to evaluate interventions to improve financial access to healthcare in sub-Saharan Africa. The use of several control outcomes and the concordance of the results across outcomes reinforce the robustness of the results.^25^

Compared with non-equivalent unexposed controls, non-equivalent dependent variables have the advantage of controlling for local history bias and selection bias.^25^ For example, if the composition of the population that uses a health facility changes abruptly at the time of the intervention or if it experiences confounding events, ANC utilisation will also be affected. However, no additional interventions that could influence the use of facility-based delivery and/or ANC were implemented in the two districts around the dates of introduction of the two interventions.

The main challenge when using non-equivalent dependent variables remains the choice of these variables. It must be certain that these variables are not influenced by the intervention but that they would respond to other important confounders as would the outcome of interest. ‘Free delivery’ was associated with an immediate and significant increase in ANC1 and ANC2, supporting the hypothesis that this intervention may lead to better use of ANC by pregnant women who wish to give birth in health facilities. This bias would have led to an underestimation of the true effects of the interventions, and therefore, would not change our conclusions.

Several authors have questioned the quality of routine data from HISs in low-income countries.^37 38^ However, in Burkina Faso, previous studies have attested to the reliability of routine data, particularly those concerning maternal health.^32^ In addition, we used primary data for deliveries, and a comparison of that data with HIS data showed that the latter has very good reliability.

In general, studies^4 5 8 31 37^ that have evaluated health interventions in sub-Saharan Africa have assumed linear—or, at most, quadratic—preintervention and postintervention trends. Our results suggest that these trends could vary frequently and that a linear approach may be insufficient to capture the real evolution of outcomes, as some authors have pointed out,^23^ and could thus lead to a biased estimation of the effects of interventions. This non-linearity of effects is consistent with the literature indicating that implementation of these interventions was not uniform over time, with phases of drug shortages, insufficient funding, etc,^20–22^ which may correspond to periods in which effects are less visible.

Another threat to internal validity in time-series studies is instrumentation bias.^25^ In both districts, data were recorded in the same way and in the same types of registers before and after the intervention for both deliveries and ANC, which allowed us to rule out any risk of instrumentation bias.

Our results confirm that interventions to improve financial access to healthcare were associated with a sustainable increase in facility-based deliveries in Burkina Faso. However, the effects were not linear over time. The results also suggest that the “free delivery” intervention, despite being local, had larger effects than the partial national subsidy. This finding is an argument in favour of the current policy of free care for women and children under 5 in Burkina Faso.

## Data Availability

Data are available on reasonable request. The datasets used and/or analysed during the current study are available from the corresponding author on reasonable request.
