# Supplementary material for: Effect of cost-reduction interventions on facility-based deliveries in Burkina Faso: a controlled interrupted time-series study with multiple non-equivalent dependent variables
Source: J Epidemiol Community Health. 2022 Dec 20;77(3):133–9. doi: 10.1136/jech-2022-218794 (PMC9933164; doi:10.1136/jech-2022-218794)
Supplement: Supplementary data [file jech-2022-218794supp001.pdf]

Table S1: Number of missing data before and after the free delivery intervention in Kaya and the national subsidy for deliveries in Zorgho.

	Kaya Health District				Zorgho Health District				
	Deliveries	ANC1	ANC2	ANC3	Deliveries	ANC1	ANC2	ANC3	ANC4
Number of health facilities	41	41	41	41	48	48	48	48	48
Number of health facilities without missing data	37	18	25	23	45	22	26	22	15
Number of observations expected in pre-policy period	564	564	564	564	767	767	767	767	767
Number of missing Data in pre-policy period	01	07	08	05	01	04	06	05	05
Percentage of missing data in pre-policy period	0.2	1.2	1.4	0.9	0.1	0.5	0.8	0.7	0.7
Number of observations expected in post-policy period	1,673	1,673	1,673	1,673	3,450	3,450	3,450	3,450	3,450
Number of missing Data in post-policy period	06	34	23	22	02	45	32	41	67
Percentage of missing data in post-policy period	0.4	2.0	1.4	1.3	0.06	1.3	0.9	1.2	1.9
Total number of observations expected	2,237	2,237	2,237	2,237	4,217	4,217	4,217	4,217	4,217
Total number of missing Data	07	41	31	27	03	49	38	46	72
Percentage of missing Data in study period	0.3	1.8	1.4	1.2	0.07	1.2	0.9	1.0	1.7

NB: Missing data included outliers excluded for analysis.
