# Supplementary material for: Effect of cost-reduction interventions on facility-based deliveries in Burkina Faso: a controlled interrupted time-series study with multiple non-equivalent dependent variables
Source: J Epidemiol Community Health. 2022 Dec 20;77(3):133–9. doi: 10.1136/jech-2022-218794 (PMC9933164; doi:10.1136/jech-2022-218794)
Supplement: Supplementary data [file jech-2022-218794supp002.pdf]

BMJ Publishing Group Limited (BMJ) disclaims all liability and responsibility arising from any reliance placed on this supplemental material which has been supplied by the author(s)

Table S2. Estimated regression coefficients and standard errors (SE) from a spline linear regression: The effects of two cost-reduction interventions on facility-based deliveries in Burkina Faso with different nonequivalent dependent variables as control outcomes.

**Coefficients**	**Deliveries**	**ANC1**	**ANC2**	**ANC3**	**ANC4**
Kaya Health District					
Baseline level (Intercept)	9.66*** (2.23)	33.19*** (3.62)	23.63**** (2.66)	10.77*** (1.97)	
Pre-intervention trend (January 2006 to May 2007)	0.47*** (0.06)	0.47*** (0.06)	0.47*** (0.06)	0.47*** (0.06)	
Immediate intervention effect	-0.32 (0.95)	10.28*** (2.60)	5.04*** (1.52)	2.77 (1.81)	
Post-intervention trends					
June to December 2007 trend	0.75*** (0.18)	-1.64*** (0.31)	-0.48* (0.21)	-0.03 (0.23)	
January 2008 to December 2009 trend	0.19***(0.03)	0.19*** (0.03)	0.19*** (0.03)	0.19*** (0.03)	
January to December 2010 trend	0.06 (0.08)	-0.69*** (0.14)	-0.63*** (0.13)	-0.59*** (0.10)	
Zorgho Health District					
Baseline level (Intercept)	11.43*** (1.70)	26.19*** (1.74)	19.76*** (1.73)	9.24*** (1.71)	1.09 (1.72)
Pre-intervention trend (January 2005 to December 2006)	0.16*** (0.02)	0.16*** (0.02)	0.16*** (0.02)	0.16*** (0.02)	0.16*** (0.02)
Immediate intervention effect	-0.13 (0.59)	-0.13 (0.59)	-0.13 (0.59)	-0.13 (0.59)	-0.13 (0.59)
Post-intervention trends					
January to December 2007 trend	0.60*** (0.07)	0.08 (0.09)	0.29** (0.08)	0.40*** (0.08)	0.14 (0.08)
January to December 2008 trend	-0.04 (0.07)	-0.04 (0.09)	-0.007 (0.08)	0.15* (0.07)	0.30*** (0.08)
January to December 2009 trend	0.38*** (0.06)	0.002 (0.07)	0.06 (0.07)	0.18** (0.06)	0.24*** (0.07)
January to December 2010 trend	-0.23*** (0.03)	-0.23*** (0.03)	-0.23*** (0.03)	-0.23*** (0.03)	-0.23*** (0.03)
January to December 2011 trend	0.25*** (0.03)	0.25*** (0.03)	0.25*** (0.03)	0.25*** (0.03)	0.25*** (0.03)
January 2012 to December 2013 trend	-0.09*** (0.02)	-0.09*** (0.02)	-0.09*** (0.02)	-0.09*** (0.02)	-0.09*** (0.02)

ANC1 (first ANC visit), ANC2 (second ANC visit), ANC3 (third ANC visit), ANC4 (fourth ANC visit). The time trend is a monthly trend.
Data are shown as estimates (SEs). *$p < 0.05$, **$p < 0.01$ & ***$p < 0.001$
